# Development of a Novel Quantum Dots and Graphene Oxide Based FRET Assay for Rapid Detection of *invA* Gene of *Salmonella*

**DOI:** 10.3389/fmicb.2017.00008

**Published:** 2017-01-17

**Authors:** Jiubiao Guo, Edward W. C. Chan, Sheng Chen, Zhenling Zeng

**Affiliations:** ^1^Guangdong Provincial Key Laboratory of Veterinary Pharmaceutics Development and Safety Evaluation, College of Veterinary Medicine, South China Agricultural UniversityGuangzhou, China; ^2^Shenzhen Key Lab for Food Biological Safety Control, Food Safety and Technology Research Center, Hong Kong PolyU Shen Zhen Research InstituteShenzhen, China; ^3^State Key Lab of Chirosciences, Department of Applied Biology and Chemical Technology, Hong Kong Polytechnic UniversityHung Hom, Hong Kong

**Keywords:** *Salmonella*, *invA*, quantum dots, graphene oxide, FRET assay, rapid detection

## Abstract

A novel, rapid and simple fluorescence resonance energy transfer (FRET) based *Salmonella* specific gene, *invA*, detection system was developed, in which quantum dots (QDs) and graphene oxide (GO) worked as fluorescent donor and quencher, respectively. By measuring the fluorescence intensity signal, the *Salmonella* specific *invA* gene could be sensitively and specifically detected with a limit of detection (LOD) of ∼4 nM of the *invA* gene in 20 min. The developed system has the potential to be used for *Salmonella* detection in food and environmental samples and further developed into a platform for detection of other bacterial pathogens.

## Introduction

*Salmonella*, a major Gram-negative bacteria enteric pathogen, has evolved numerous strategies to infect and proliferate in a vast array of hosts, such as humans and animals, causing a wide range of food- and water-borne diseases (LaRock et al., 2015). It is estimated that *Salmonella* resulted in 17,000 hospitalization and 585 deaths each year in the US, causing $2.3–3.6 billion economic lost annually (Foley and Lynne, 2008). There is an urgent need for rapid and sensitive detection methods of *Salmonella*, especially methods that do not require sophisticated equipment or intensive labor, to prevent outbreaks and recalls due to *Salmonella* contamination.

*Salmonella* culture-based detection strategies are time-consuming and labor intensive, although they are the main methods for diagnosis (Okamura et al., 2008). Real-time PCR (rt-PCR; Hara-Kudo et al., 2005) methods can detect *Salmonella* by measuring the increased fluorescence via the amplification of DNA; the loop-mediated isothermal amplification (LAMP) strategy (Techathuvanan et al., 2011) which relies on autocycling strand displacement DNA synthesis is novel, rapid and simple, but they suffer from expensive equipment and depend on skillful technicians, etc. The last decade has witnessed a rapid development of biosensing techniques and new biomaterials (Saikia et al., 2013; Jana et al., 2015; Unser et al., 2015; Zheng et al., 2015). They have been proven to be valid for various applications ranging from pathogens detection to cancers therapies (Alocilja and Radke, 2003; Liu et al., 2009; Chen et al., 2015; Shi et al., 2015). Graphene is the first two-dimensional atomic crystal discovered, and chemically derived graphene oxide (GO) is served as a precursor for grapheme. GO is an atomically thin sheet with large surface area, possibility of easy functionalization by various functional groups, and long-range resonance energy transfer distance, which make it an ideal quencher in bioapplications (Loh et al., 2010; Novoselov et al., 2012). Recently, the semiconductor, quantum dots (QDs), is considered as one of the most promising emerging fluorescent dyes. When compared with conventional dyes, QDs display superior features such as photobleaching resistance, narrow, symmetric and size-tunable absorption and emission wavelength (Wu et al., 2003; Dong et al., 2010). Due to these interesting properties, QDs have been widely used in biological applications (Medintz et al., 2005; Lu et al., 2011; Saikia et al., 2013; Wu et al., 2015). In addition, a few studies have reported the combination usage of GO and QDs in detecting and sensing biomolecules (Liao et al., 2014). *InvA* gene, one of the virulence chromosomal genes, has been proved to be unique to *Salmonella* and can be used as a suitable PCR target for the detection of *Salmonella* (Rahn et al., 1992; Zahraei Salehi et al., 2005; Shanmugasamy et al., 2011). In addition, an *invA* targeted isothermal target and probe amplification (iTPA) approach has been applied by Kim et al. (2011) for the specific and rapid detection of *Salmonella*. But few studies present rapid and sensitive *invA* gene detection in *Salmonella* by combinational usage of GO and QDs as fluorescence resonance energy transfer (FRET) pair (Lee et al., 2015; Zhang et al., 2016).

In the present work, an assay based on FRET pair between QDs and GO technology was developed to target the highly conserved *invA* gene of *Salmonella* for the purpose of rapid and sensitive detection of this important pathogen (Zahraei Salehi et al., 2005).

## Materials and Methods

### Reagents and Materials

Graphene oxide was a kind gift from Dr. Yang’s lab (The Hong Kong Polytechnic University, HK, China). Carboxyl-modified 525nm QDs were purchased from Invitrogen, Ltd. (USA). Phosphate buffered saline (PBS) pH 7.4 and bovine serum albumin (BSA) were prepared accordingly. N-hydroxysuccinimide (NHS) and 1-ethyl-3-(3-dimethylaminopropyl) carbodiimide hydrochloride (EDC) were purchased from Sigma-Aldrich (St. Louis, MO, USA). Microcon molecular weight cut-off (MWCO) spin filters were obtained from Millipore Corporation (Bedford, MA, USA). Luria Bertani (LB) broth was purchased from Qingdao Hope Bio-Technology Co., Ltd. (China) and prepared by following supplier’s instruction. DNA extraction kits were purchased from Qiagen (Germany). A 30-mer single strand *invA* oligo (5′-CTTTCGTCTGGCATTATCGATCAGTACCAG-3′) and a 26-mer single strand control oligo (5′-GTGAAATTATCGCCACGTTCGGGCAA-3′) were extracted from the highly conserved *Salmonella typhi murium invA* gene (GenBank: M90846.1). Single-base mismatched oligo (M1: 5′-CTTTCGTGTGGCATTATCGATCAGTACCAG-3′), double-base mismatched oligo (M2: 5′-CTTTCGTGTGGCATTATCCATCAGTACCAG-3′) and the control oligo were synthesized for the specificity test of the developed system. Amine-modified capture A (5′-ATGCCAGACGAAAG/Aminolinker C7/-3′) and capture B (5′-/Aminolinker C6/CTGGTACTGATCGA-3′) that were complementary to the *invA* oligo were designed and synthesized. The primers that can specifically amplify the desired part of the *invA* gene were synthesized as well. The sequence of the forward primer is F-invA: GCCTACAAGCATGAAATGGCAGAAC and the reverse primer is R-invA: TCATCGCACCGTCAAAGGAACC. The length of the amplified product is about 649 bp. All the oligonucleotides listed in **Table 1** were synthesized by Beijing Genomics Institute (Shenzhen, Guangdong, China) and prepared according to the supplier’s instruction.

**Table 1:** The DNAsequences of synthesized oligonucleotides in this study.

Oligos/Primers	Sequence (5′-3′)
*invA* oligo	CTTTCGTCTGGCATTATCGATCAGTACCAG
Control oligo	GTGAAATTATCGCCACGTTCGGGCAA
M1	CTTTCGTGTGGCATTATCGATCAGTACCAG
M2	CTTTCGTGTGGCATTATCCATCAGTACCAG
Capture A	ATGCCAGACGAAAG/Aminolinker C7
Capture B	Aminolinker C6/CTGGTACTGATCGA
F-invA	GCCTACAAGCATGAAATGGCAGAAC
R-invA	TCATCGCACCGTCAAAGGAACC


### *InvA* Fragment Preparation

*Salmonella Typhimurium* (*S. Typhimurium*) was inoculated in LB broth and maintained at 37°C overnight. 1.5 mL of the overnight culture was used to extract genomic DNA and suspended into 50 μL distilled water with a concentration of 0.6 μg/μL. 0.5 μL of the extracted *Salmonella* genomic DNA (∼0.3 μg) was used as template for PCR reaction. 10 μL of the purified PCR products (100 ng/μL) were used for the following detection analysis.

### Conjugation of GO and QDs with Probes

The process of conjugation between GO or QDs with capture A or B was realized by EDC/NHS assisted covalent bonding where EDC [1-Ethyl-3-(3-dimethylaminopropyl)-carbodiimide] is a zero-length cross-linking agent used to couple carboxyl or phosphate groups to primary amines and NHS (hydroxysuccinimide) was used as a stabilizer. The conjugation between GO and capture A was performed as described in a previous study where capture A was derived from the positive strand of and complementary to *Salmonella invA* gene (Shi et al., 2015). Briefly, freshly prepared NHS (5 mM) and EDC (1 mM) were added into the GO solution (5 mg/mL), vortex for 2 min and sonication for 15 min. Then the treated GO was mixed with 30 μM capture A, sonicated at room temperature (RT) for 1 h. The generated GO-capture A conjugate was further purified and washed by DI-H_2_O by centrifugation at RT at 10,000 rpm for several times. In order to prevent unspecific binding of QDs with GO-capture A, the GO-capture A conjugate was further treated with 0.5 mg/mL BSA at RT for 30 min and then rinsed with DI-H_2_O.

For the conjugation of QDs with capture B, which was derived from positive strand of and complementary to *Salmonella invA* gene, most of the procedures were carried out following the manufactures’ instructions, but with modifications. 50 μl QDs stock solution (8 μM) was diluted in 1xPBS (pH7.4), mixed with 20 μM capture B, followed by immediate addition of EDC (1 mg/mL). The mixture was then mixed by rotating at RT at dark environment for 2 h. The unbound capture B was removed by using a centrifugal filter and then washed by 1xPBS (pH7.4) for several times. 0.1% N_3_Na was added in the final products and stored at 4°C in dark environment for future use.

### Characterization

Fourier transform infrared spectrum (FT-IR) spectra of GO, GO-capture A, QDs and QD-capture B were measured with a PerkinElmer Spectrum 100 FT-IR spectrometer (PerkinElmer Inc., USA). Zeta potentials of GO, GO-capture A, QDs and QD-capture B were characterized by a ZetaPlus Zeta Potential Analyzer (Brookhaven Instruments Corp., USA).

### Fluorimetric Assay

In the system, the *invA* oligo worked as a bridge to bring the GO-capture A and QD-capture B conjugates close enough by being complementary to both capture A and B, the energy emitted by QDs would be quenched by GO in the form of decreased fluorescence intensity. 0.5 mg/mL BSA passivated GO-capture A (60 μg/mL) was first mixed with *invA* oligo and incubated at 55°C for 10 min, then a desired concentration of QD-capture B (150 nM) was added, the mixture was incubated at 55°C for another 10 min. The total reaction volume was 50 μl. The fluorescence intensity was measured by using a Cary Eclipse Fluorescence Spectrophotometer (Agilent Technologies, USA) with the excitation wavelength set as 320 nm and emission range as 480–580 nm. For the specificity test of the system, the *invA* oligo was replaced either by M1 or M2 or control oligo in the reaction mixture, and for the application assay of the developed system, the relative PCR product was added in the place of *invA* oligo. All the assays were repeated for at least five times.

### Statistical Analysis

Standard Error of the Mean (SEM) was used to express the error in replicates. For group comparisons, the statistical test used was unpaired two-tailed *t*-test (*P* < 0.05). The statistical analyses were performed with GraphPad Prism 5 (GraphPad Software, Inc., USA).

## Results and Discussion

### Principal of the *Salmonella invA* Gene Detection System

**FIGURE 1:**
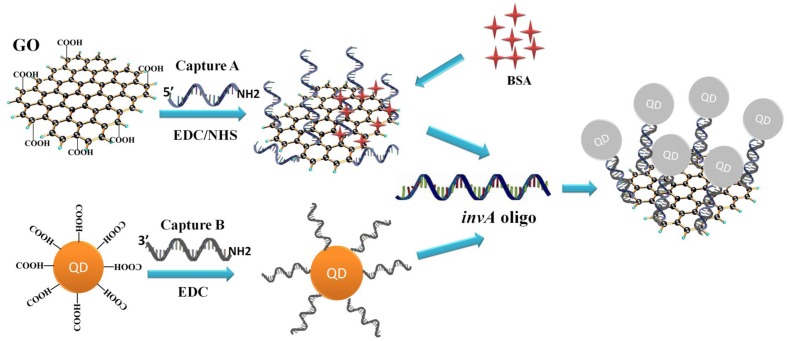
**Principle of GO-QDs FRET biosensor.** In the system, carboxyl QDs (donor) and GO (quencher) were first conjugated with the capture probes B and A, respectively, with the aid of EDC/NHS. Upon the addition of the complementary *invA* oligo of *Salmonella*, the QD and GO conjugates could be brought into close proximity to make the FRET pair work, the energy emitted from excited QDs would be quenched by GO.

The *invA* gene of *Salmonella* is highly conserved and has been used as a target for the detection of *Salmonella* previously (Zahraei Salehi et al., 2005). The principle of the developed *Salmonella invA* gene detection system is illustrated in **Figure 1**. In the system, carboxyl QDs (donor) and GO (quencher) were first conjugated with the capture B and A, respectively, with the aid of EDC/NHS. Upon the addition of the complementary *invA* oligo of *Salmonella*, the QDs and GO conjugates could be brought into close proximity to make the FRET pair work, the energy emitted from excited QDs would be quenched by GO. Based on this mechanism, the *invA* gene of *Salmonella* can be detected via the measurement of the fluorescence intensity change in the developed system.

### Characterization of GO and QDs

After conjugation of GO and QDs with the corresponding oligonucleotides, the conjugates of GO-capture A and QD-capture B were analyzed by zeta potentials. When compared with that of GO, the zeta potential value of GO-capture A was about -25 mV, much closer to that of capture A alone, and both zeta potential peaks overlapped (**Supplementary Figure S1A**); so did the QD-capture B zeta potential value, which was about -12.5 mV, closer and overlapped with that of capture B alone as well (**Supplementary Figure S1B**), indicating that the surface of GO and QDs were covered by the corresponding captures after conjugation. In addition, the successful conjugation between GO and capture A or QDs and capture B were confirmed by FTIR spectra analysis as shown in **Supplementary Figure S2**. The characteristic amide vibration absorption peak could be obviously detected at around 1655 cm^-1^ both in GO-capture A (**Supplementary Figure S2A**) and QD-capture B (**Supplementary Figure S2B**), suggesting that an amide bond has formed between the carboxyl group on GO or QDs and the amine group of oligonucleotides.

**FIGURE 2:**
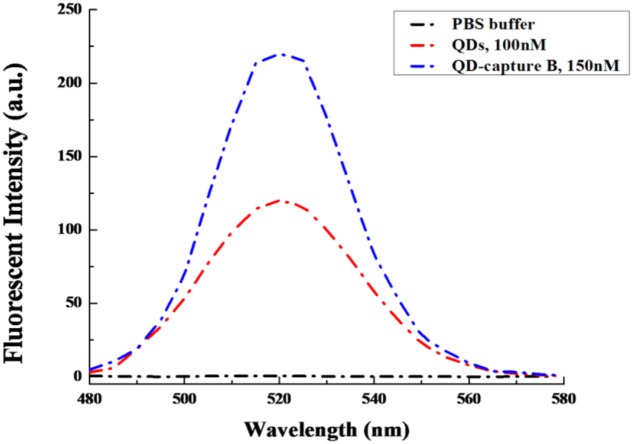
**The emission spectra comparison between QDs and QD-capture B conjugate.** The QD and QD-capture B conjugate were diluted in 1xPBS, pH = 7.4. The excitation wavelength was set at 320 nm.

Moreover, in order to check whether the capture B oligo could alter the emission pattern of QDs or not under the excitation wavelength of 320 nm, the emission spectra of QDs and QD-capture B were compared, and both of them were found to display very similar emission patterns with the emission peak at about 520 nm (**Figure 2**).

### FRET Biosensor for *invA* Detection

**FIGURE 3:**
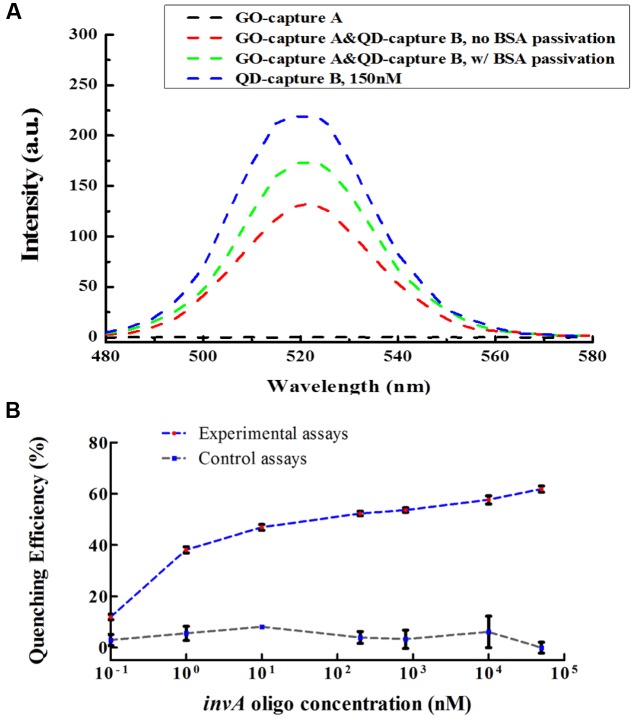
**The BSA passivation effect and quenching efficiency of the developed *invA* gene biosensor.** **(A)** The BSA passivation effect in decreasing the non-specific adsorption between GO and QD conjugates. Briefly, in order to check the BSA passivation effect in preventing unspecific binding between QD and GO conjugates, the GO-capture A conjugate was further treated with or without 0.5 mg/mL BSA at RT for 30 min and then rinsed with DI-H_2_O, then the fluorescence intensity was measured. The data were analyzed by OriginPro 8.5. **(B)** For the quenching efficiency assays, in 50 μl reaction volume, BSA passivated GO-capture A (60 μg/mL) was first incubated with serially diluted *invA* oligo at 55°C for 10 min, then 150 nM QD-capture B was added to the reaction mixture and incubated at 55°C for another 10 min. The fluorescence intensity was measured under 320 nm excitation wavelength and the values at 520 nm were extracted for the calculation of quenching efficiency. The only difference between the experimental assays and the control assays was that the BSA passivated GO-capture A which was included in the experimental assays was replaced by BSA passivated GO (without capture A) in the control assays. The SEM (Standard Error of the Mean) error bars were calculated from at least three replicates. The data were analyzed by GraphPad Prism.

**FIGURE 4:**
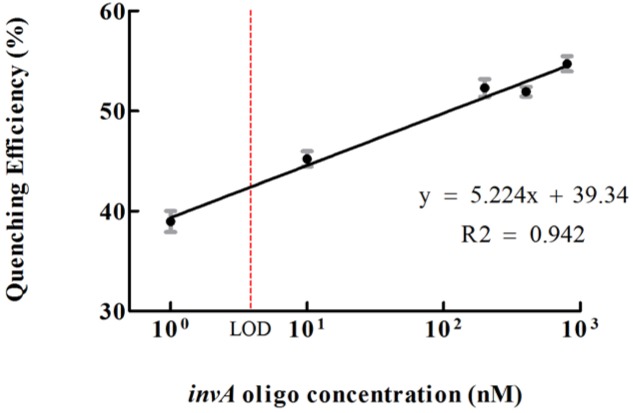
**The limit of detection (LOD) of the developed *invA* gene biosensor.** The quenching efficiencies which were calculated as according to the listed formula were potted versus different concentrations of *invA* oligo. LOD = 3^∗^S/k (*S* means standard deviation of negative control, *k* means slope).The SEM (Standard Error of the Mean) error bars were calculated from at least three replicates.

Graphene oxide has relatively large surface area for binding but does not exhibit binding specificity. In order to reduce or avoid undesired interaction between GO and QD conjugates, the GO conjugates were first passivated by BSA. After passivation, the non-specific interaction between GO and QD conjugates could be decreased but could not be completely avoided (**Figure 3A**). To investigate the quenching efficiency of developed biosensor in the detection of *invA* gene, experimental and control assays were carried out. In the experimental assays, various concentration of synthesized *invA* oligo was studied with the passivated GO-capture A concentration fixed at 60 μg/mL, in the control assays, the only difference was that the passivated GO-capture A was replaced by the same concentration of passivated GO (without capture A). Theoretically, before saturation, the higher concentration of the *invA* oligo included in the reaction mixture, the higher quenching efficiency would be observed. The formula of quenching efficiency is Q = (F0 - Fq)/F0^∗^100%, in which the *F*_0_ represents the fluorescence intensity of QD-capture B before quenching and the *F*_q_ means that after quenching. About 50% quenching efficiency could be detected with as low as 10 nM *invA* oligo, but the quenching efficiency was not increased significantly with the higher concentration of *invA* oligo. For comparison, in the control assays, almost no quenching efficiency could be detected (**Figure 3B**). The limit of detection (LOD) of the present system in detecting *invA* gene of *Salmonella* was further determined which was ∼4 nM (**Figure 4**).

**FIGURE 5:**
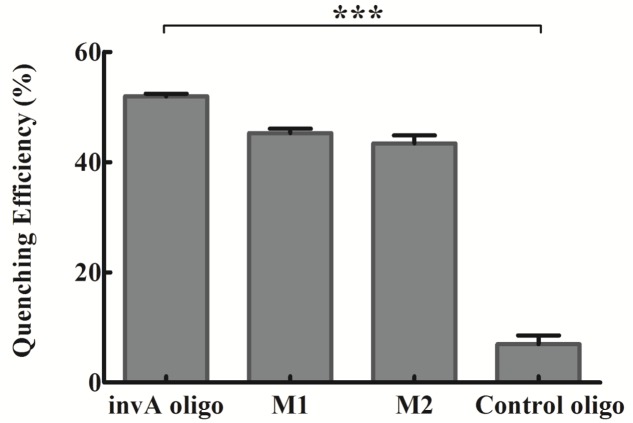
**The specificity of the developed *invA* gene biosensor.** The concentrations of *invA* oligo (fully complementary to probes), M1 (one-base mismatch), M2 (two-base mismatch) and control oligo (not complementary to neither probes) used were fixed at 400 nM. The SEM (Standard Error of the Mean) error bars were calculated from at least three replicates. The difference between *invA* oligo and control oligo groups was significant (*P* < 0.0001, two tail *t*-test), but the difference between *invA* oligo and M1 or M2 groups were not significant (*P* > 0.05, two tail *t*-test). The data was analyzed by GraphPad Prism. ^∗∗∗^*P* < 0.0001.

The biosensor specificity in detecting *invA* gene of *Salmonella* was analyzed by using mismatched oligonucleotides and control oligo. The quenching efficiency of 400 nM *invA* oligo was ∼52%, and that of 400 nM M1 and M2 were ∼45 and ∼43%, respectively, while that of control oligo was smaller than 10%, a significant difference from *invA* oligo (*P* < 0.0001, two-tailed *t*-test; **Figure 5**), suggesting that the system is very specific for detecting *invA* gene, while its discrimination power is not very high when the oligo is within couple of nucleotide difference from *invA* gene. Consistently, a higher selectivity was reported by applying similar detection approaches (Yang et al., 2008; Shi et al., 2015). In addition, J.S. Kim and colleagues witnessed very high *Salmonella* spp. detection specificity by using *invA* gene as target, with all of 10 *Salmonella* spp. could be specifically detected, but not the 40 non-*Salmonella* strains (Kim et al., 2011).

### The Possible Application of the Developed *invA* Gene Detection Biosensor

**FIGURE 6:**
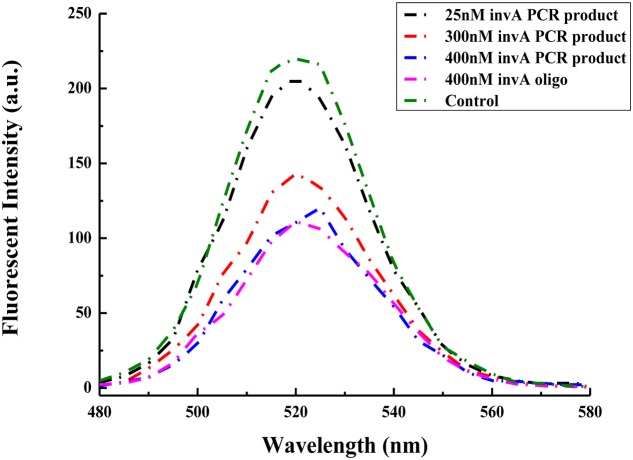
**Comparison between the ability of the *invA* gene PCR product and synthesized *invA* oligo in mediating changes in fluorescence intensity of the developed biosensor.** In the control sample, only GO-capture A and QD-capture B were included, no *invA* gene PCR product or *invA* oligo was added to the reaction mixture.

The *invA* gene has previously been used as target to specifically detect *Salmonella* spp. in food samples with high sensitivity (Kim et al., 2011). In order to check the possibility of the present biosensor in detecting the *invA* gene from *Salmonella* of environmental samples, specific primers were used to amplify the *invA* gene which cover the complementary fragment of probes that are conjugated onto GO and QDs. Compared with the same concentration of the *invA* oligo, 400 nM PCR product of *invA* gene could cause almost same degree of decrease in fluorescent intensity, and the fluorescent intensity varied accordingly with the change of the concentrations of PCR product of *invA* gene. The fluorescence intensity pattern from using 25 nM PCR product was almost the same as that of control assay, and for the assay with 300 nM PCR product, the intensity peak (around 520 nm) was about half of that from the control assay (**Figure 6**), indicating that the present biosensor has the potential to be applied in detecting the *invA* gene of *Salmonella* in food or environmental samples.

## Conclusion

*Salmonella* infections continue to be a major public health threat worldwide. To achieve efficient and timely prevention of *Salmonella* outbreaks, new detection methods featured by rapidness, high sensitivity and simplicity of operation are prerequisite. In the present study, a novel *Salmonella* detection system based on the highly conserved *invA* gene was developed by utilizing nanomaterials of QDs and GO and the FRET technology. After careful measurement and evaluation, the reported system could specifically detect as low as ∼4 nM *invA* gene of *Salmonella*. The use of QDs to pair with GO could significantly improve the sensitivity and signal stability and could be potentially applied for in-field *Salmonella* detection to ensure food safety. Further study will be carried out to investigate the feasibility and detection efficiency of the developed biosensor in detecting *Salmonella* spp. directly in food samples, and the convenience for in-field *Salmonella* detection in the near future.

## Author Contributions

JG designed, conducted the experiments, analyzed the data, and wrote the manuscript. EC designed the experiment and edited the manuscript. SC and ZZ initiated and supervised the project, and edited the manuscript.

## Conflict of Interest Statement

The authors declare that the research was conducted in the absence of any commercial or financial relationships that could be construed as a potential conflict of interest.
