# Supplementary material for: Development of a Novel Quantum Dots and Graphene Oxide Based FRET Assay for Rapid Detection of invA Gene of Salmonella
Source: Front Microbiol. 2017 Jan 17;8:8. doi: 10.3389/fmicb.2017.00008 (PMC5239778; doi:10.3389/fmicb.2017.00008)
Supplement: Supplementary file 1 [file Data_Sheet_1.DOCX]

**Development of a novel quantum dots and graphene oxide based FRET assay for rapid detection of *invA* gene of *Salmonella***

Jiubiao Guo^1,2^, Edward Wai Chi Chan^2,3^, Sheng Chen^2,3^*, Zhenling Zeng^1^*

^1^Guangdong Provincial Key Laboratory of Veterinary Pharmaceutics Development and Safety Evaluation, College of Veterinary Medicine, South China Agricultural University, Guangzhou, China

^2^Shenzhen Key lab for Food Biological Safety Control, Food Safety and Technology Research Center, Hong Kong PolyU Shen Zhen Research Institute, Shenzhen, P. R. China

^3^State Key Lab of Chirosciences, Department of Applied Biology and Chemical Technology, The Hong Kong Polytechnic University, Hung Hom, Kowloon, Hong Kong

*To whom correspondence may be addressed:

Prof. Zhenling Zeng

zlzeng@scau.edu.cn

Prof. Sheng Chen

sheng.chen@polyu.edu.hk.

**Keywords:** *Salmonella*, *InvA*, quantum dots, graphene oxide, FRET assay, rapid detection

**Supplementary Materials**

**Zeta potential analysis**

Zeta potentials of GO, GO-capture A, QD and QD-capture B were characterized by a ZetaPlus Zeta Potential Analyzer (Brokhaven Instruments Corp., USA) in DI water at pH=7.0.

**
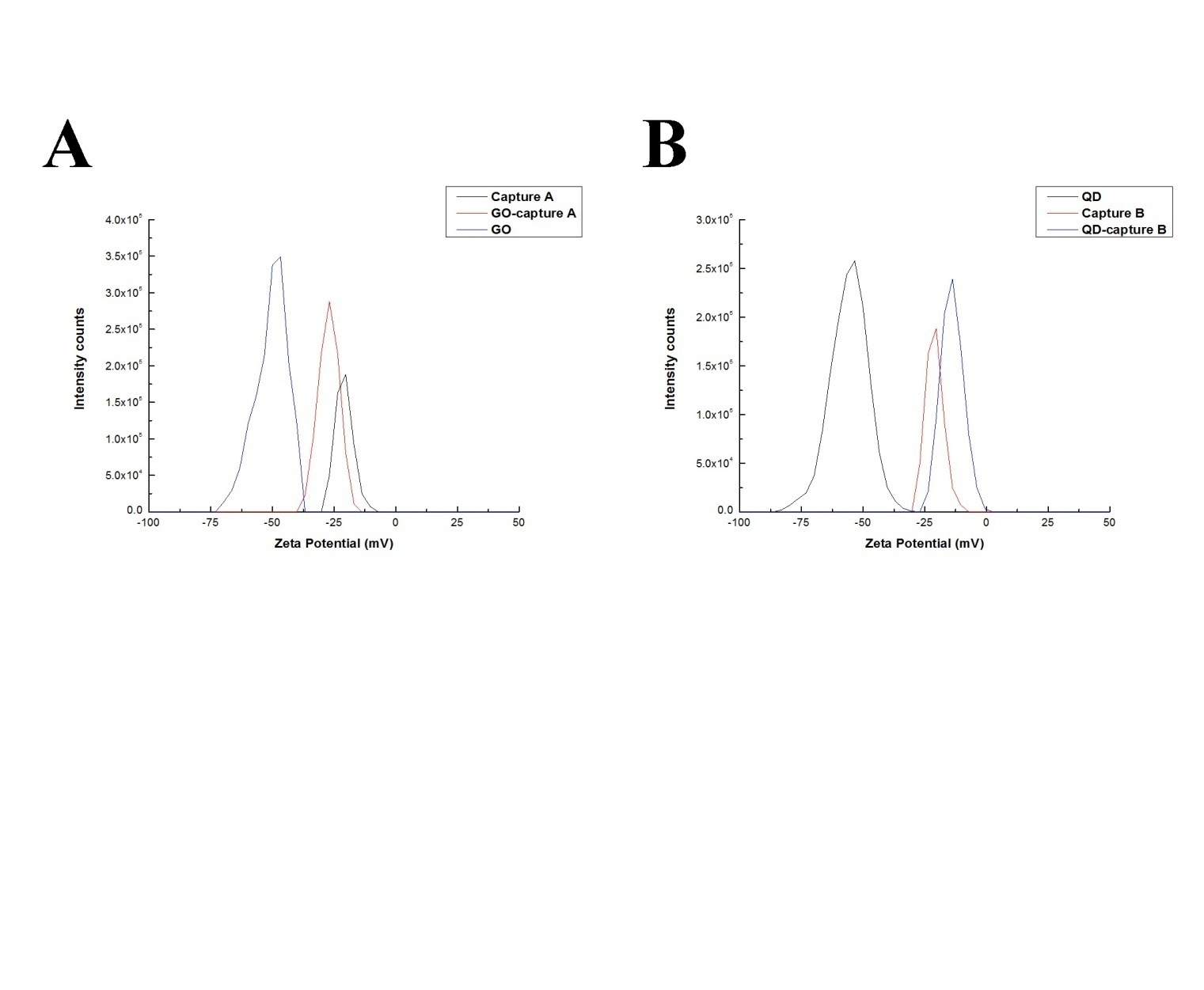
**

**Figure. S1** Zeta potential measurements of (A) GO&GO-capture A and (B) QD&QD-capture B.

**FTIR spectra measurements**

Fourier transform infrared spectrum (FT-IR) spectra of GO, GO-capture A, QD and QD-capture B were measured with a PerkinElmer Spectrum 100 FT-IR spectrometer (PerkinElmer Inc., USA).


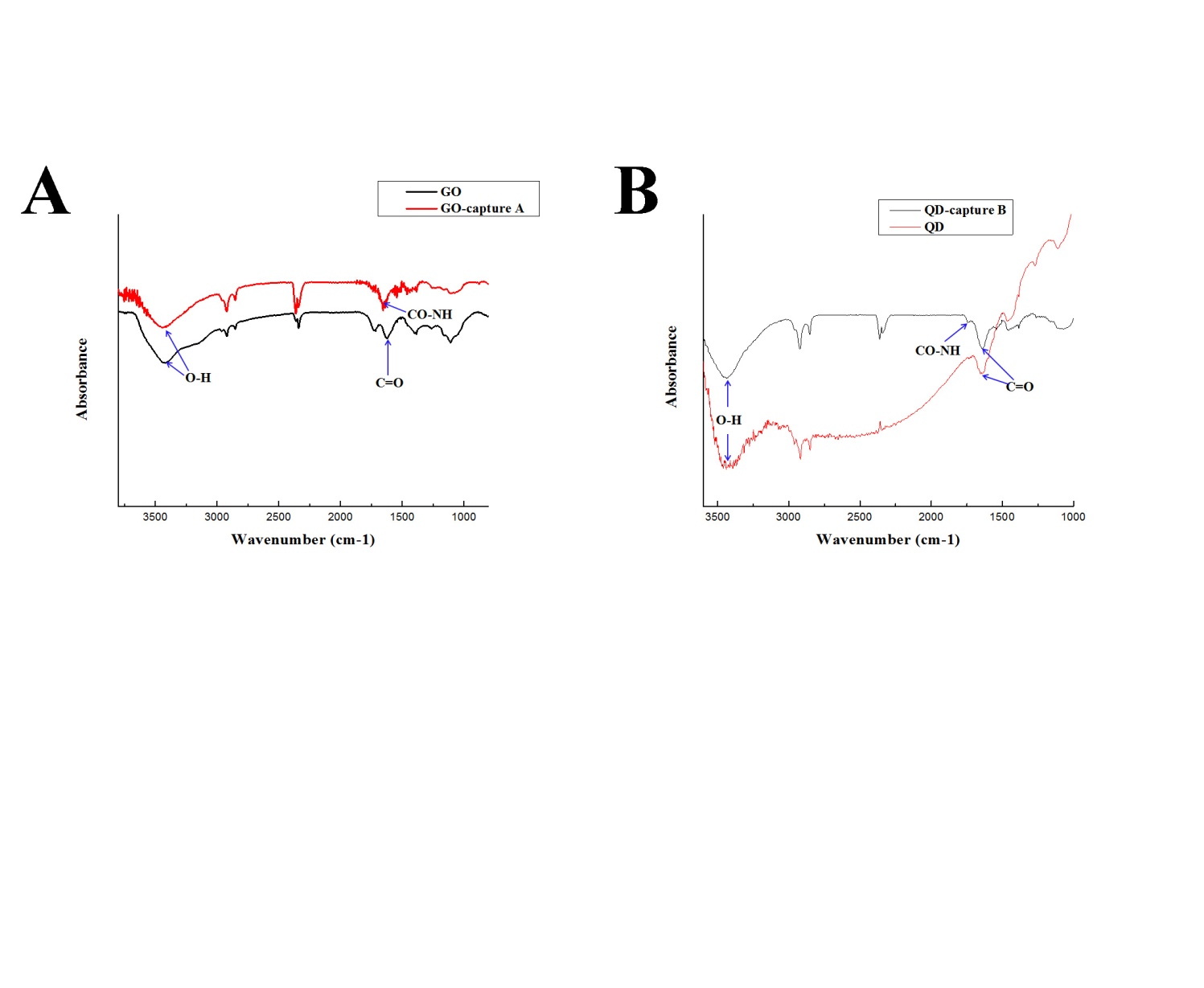


**Figure. S2** FTIR analysis of (A) GO&GO-capture A and (B) QD&QD-capture B.
